# From Retigabine to Azetukalner: Reviving Voltage‐Gated Potassium Channels for Epilepsy Therapy

**DOI:** 10.1002/ardp.70279

**Published:** 2026-07-03

**Authors:** Pascal Rosendahl, Lukas Schulig, Andreas Link

**Affiliations:** ^1^ Department of Pharmaceutical/Medicinal Chemistry, Institute of Pharmacy University of Greifswald Greifswald Germany

**Keywords:** azetukalner, clinical studies, drug development, KCNQ, KV7, retigabine

## Abstract

Heterotetrameric K_V_7.2/3 potassium channels were retrospectively identified as the target of the analgesic flupirtine and the antiepileptic retigabine (ezogabine). Clinical utility of these agents ended after decades or 6 years, respectively, before their full scope was explored. Market withdrawals in 2017 and 2018 hampered research in other medical fields and left a gap for researchers using these compounds off‐label in experimental indications and for patients with K_V_7.2/3 malfunctions. Failure of these drugs might be regarded as a reason to abandon this class of compounds, due to the notion that toxicity might be a class effect and to competitive markets. Yet, evidence accumulated that the toxicity of both compounds is not an inherent property of modulators of K_V_7.2/3 channels, but rather the result of an oxidation‐sensitive metabophore/toxicophore. Second, epilepsy associated with K_V_7.2/3 channelopathies might rationally be addressed best with modulators of this validated drug target. Employing retrometabolic drug design to remodel the enzyme‐labile carbamate structure and the central highly substituted ring, azetukalner (formerly encukalner, XEN1101) emerged from the unlucky forerunners. This structural analog of retigabine exhibits improved metabolic stability, increased potency, and enhanced blood–brain barrier penetration compared with its predecessor. This review details the synthesis, physicochemical properties, and clinical results of azetukalner.

## Graphical abstract

From the withdrawal of retigabine to the breakthrough of azetukalner, this review explores how rational redesign of K_V_7.2/3 potentiators overcame toxicity hurdles. We detail the synthesis, improved metabolic stability, and robust clinical outcomes in epilepsy, highlighting a new era of targeted potassium channel therapy.

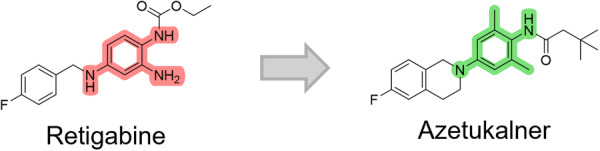

## 1 Introduction

**Figure 1:**
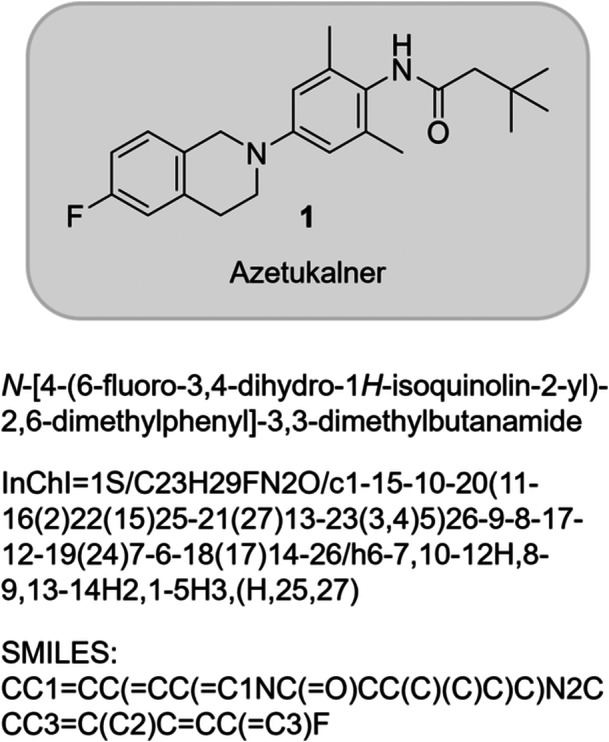
Chemical structure of azetukalner, including different chemical identifiers.

Azetukalner (Figure 1) is a potent, selective positive allosteric modulator of neuronal K_V_7.2–7.5 potassium channels, particularly K_V_7.2, K_V_7.3, and their heteromers. By stabilizing the open state of these channels, it promotes a hyperpolarized resting membrane potential, thereby reducing rapid action potential spiking.

K_V_7 channels, encoded by *KCNQ1–5* genes, assemble as tetramers consisting of a voltage‐sensing domain (S1–S4) and a pore domain (S5–S6). Together, they generate the M‐current, a voltage‐activated, slowly activating, and noninactivating current that acts as a “brake” against hyperexcitability in both the central and peripheral nervous systems [1, 2].

In the heart, K_V_7.1 generates the slowly activating delayed rectifier current, with mutations causing hereditary arrhythmias and QT prolongation [1]. In the nervous system, K_V_7 channels, particularly K_V_7.2/3, play a critical role in controlling neuronal excitability and modulating seizure susceptibility [3]. Loss‐of‐function, as well as gain‐of‐function variants in *KCNQ2* are associated with a spectrum of epilepsy phenotypes, ranging from self‐limited familial neonatal seizures to severe developmental and epileptic encephalopathies [4].

The clinical history of K_V_7 openers is marked by significant safety challenges. Flupirtine, used for pain management, was withdrawn from several markets in 2018 due to severe hepatotoxicity [5, 6]. Similarly, retigabine, approved for partial‐onset seizures in 2011, was discontinued in 2017 following reports of serious skin and mucosal pigmentation [7, 8, 9].

Beyond pain management and epilepsy, the modulation of the M‐current holds therapeutic potential for stroke, traumatic brain injury, drug addiction, and mood disorders [2]. Other K_V_7.2/3 activators in development include opakalim (BHV7000) [10], pynegabine (HN37) [11], QO‐83 [12], and CB‐003 [13], plus recently repurposing of the D2‐receptor antagonist JNJ‐37822681 [14], though comprehensive clinical efficacy data for these agents remain limited.

Azetukalner represents a recent effort to optimize the K_V_7 opener profile. It has reached phase III clinical trials for focal seizures and is currently under investigation in phase III studies for primary generalized tonic clonic seizures, major depressive disorder, and bipolar depression. Preliminary data suggest that azetukalner is effective in treating drug‐resistant focal seizures and maintains a favorable safety profile, notably lacking the bluish tissue discoloration associated with its predecessors [3].

## 2 Historical Background and Origin of the Pre‐Stem ‐kalner

Originally described in a patent by Valeant Pharmaceuticals in 2008, azetukalner was acquired by First‐Order Pharmaceuticals in 2015 and later obtained by Xenon Pharmaceuticals in 2017. It was formerly known as encukalner, XEN1101, 1OP‐2198, or VRX621698 [15].

Pharmacologically, azetukalner may be viewed as a successor to retigabine. However, this relationship is obscured by their disparate stems (‐gabine vs. ‐kalner). Retigabine was erroneously assigned the ‐gabine suffix based on early, incorrect assumptions that it targeted GABA receptors. Subsequent research revealed its primary mechanism as the direct opening of K_V_7channels [16]. This misnomer highlights a persistent gap in drug nomenclature. Similarly, the name azetukalner was inspired by flindokalner, sharing the suffix ‐kalner, combining Kalium for potassium and ‐ner for neuronal activity. However, the use of this suffix is itself ambiguous. While flindokalner was introduced as a calcium‐activated potassium (Maxi‐K) channel opener, it was later described as a K_V_7 opener, illustrating the lack of precision in current naming conventions [17, 18]. These examples underscore an ambiguity in the guidelines for International Nonproprietary Names and US Adopted Names (USAN). While the World Health Organization introduced ‐kalner for Maxi‐K openers, its application across different channel types risks inconsistency. A parallel is seen with the ‐kalim stem, which shifted from specifying potassium channel activators (formerly antihypertensive) to broadly signifying nonspecific openers, such as opakalim (BHV7000) [19, 20, 21]. Given the rise of selective K_V_7modulators, regulators should refine the ‐kalner nomenclature to ensure naming aligns with pharmacological precision and avoids clinical confusion.

To date, azetukalner is registered in 15 clinical trials, including 13 unique studies plus two open‐label extensions (OLEs) of their respective programs. The program is most advanced in focal epilepsy, where the Phase III X‐TOLE2 was recently completed, and another Phase III trial, X‐TOLE3, is currently recruiting. The major depressive disorder (MDD) program recently entered Phase III with X‐NOVA2 and X‐NOVA3, building on a completed Phase II study published in 2025. Other new indications include primary generalized tonic clonic seizures (X‐ACKT) or bipolar depression (X‐CEED) with Phase III clinical trials currently recruiting.

All active trials are sponsored by Xenon Pharmaceuticals except for one study, NCT04827901, sponsored by the National Institute of Mental Health, which was an independent academic investigation. This listing is based on ClinicalTrials.gov searches; additional country‐specific registrations may exist outside this database [22, 23, 24, 25, 26, 27, 28, 29, 30, 31, 32, 33, 34, 35, 36].

## 3 Synthesis of Azetukalner

The synthetic pathway for azetukalner was first described in 2008 in a patent of Valeant Pharmaceuticals entitled “Derivatives of 4‐(*N*‐azacycloalkyl)anilides as potassium channel modulators” (WO 2008/024398) [37].

**Scheme 1:**
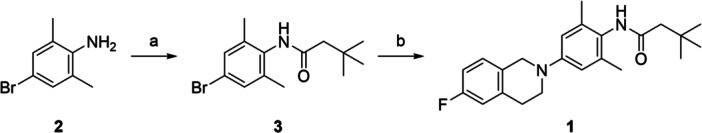
Synthetic route to azetukalner (**1**) from commercially available 4‐bromo‐2,6‐dimethylaniline (**2**), adapted from WO 2008/024398 [31]. (a) Amide formation: 3,3‐dimethylbutanoyl chloride, TEA, ACN, rt, 4 h. (b) Pd‐catalyzed C–N cross‐coupling: 6‐fluoro‐1,2,3,4‐tetrahydroisoquinoline, Pd(dba)2, DavePhos, *t*‐BuOK, dry toluene, 90°C, 16 h.

Azetukalner can be synthesized in two key steps starting from 4‐bromo‐2,6‐dimethylaniline (**2**) (Scheme 1), a commercially available compound that can alternatively be prepared by bromination of the corresponding aniline with reagents such as *N*‐bromosuccinimide in an aprotic solvent like acetonitrile (ACN). Following bromination, the product is typically purified by filtration through Celite or via flash chromatography.

Step 1: Amide Formation

4‐Bromo‐2,6‐dimethylaniline (**2**) reacts with 3,3‐dimethylbutanoyl chloride (**3**) in ACN in the presence of triethylamine (TEA) as a base. The reaction proceeds at room temperature (rt) for approximately 4 h, yielding *N*‐(4‐bromo‐2,6‐dimethylphenyl)‐3,3‐dimethylbutanamide (**3**) as a crystalline solid, which is isolated by filtration.

Step 2: Palladium‐Catalyzed Cross‐Coupling

The bromo intermediate **3** undergoes a Pd‐catalyzed C–N coupling with 6‐fluoro‐1,2,3,4‐tetrahydroisoquinoline. The reaction employs bis(dibenzylideneacetone)palladium(0) (Pd(dba)_2_) as the palladium source, 2‐(dicyclohexylphosphino)‐2‐(dimethylamino)biphenyl (DavePhos) as the bulky phosphine ligand, and potassium *tert*‐butoxide as a strong base. The coupling is performed in dry toluene under an inert atmosphere, with heating at 80°C for 16 h. After completion, the product is purified by recrystallization from toluene to afford azetukalner (**1**).

## 4 Advances Compared to Retigabine and Flupirtine

**Scheme 2:**
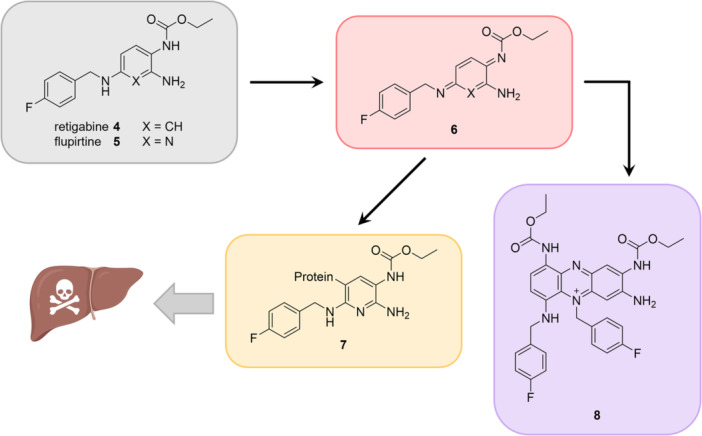
Metabolic activation of **4** and **5** via oxidation leading to the formation of reactive quinone diimine metabolites **6**, a process linked to the generation of bluish phenazinium species **8** and covalent protein adducts **9**, contributing to their observed hepatotoxicity.

Flupirtine (**5**) and retigabine (**4**) share a common structural foundation, a triaminoaryl scaffold. This shared metabophore renders both compounds vulnerable to metabolic oxidation, thereby forming reactive quinone diimine intermediates (Scheme 2) [38].

The main concern with flupirtine (**5**) was the potential to induce idiosyncratic drug‐induced liver injury (DILI). The underlying mechanism of this hepatotoxicity involves both metabolic activation and immune‐mediated pathways. Specifically, the formation of reactive quinone diimine metabolite **6** (X═N) is believed to be critical. Genetic polymorphisms in key enzymes, including NAT2, UGT1A1, and GST, influence the balance between toxic quinone diimine formation and detoxification pathways [39, 40]. However, a patient stratification strategy to avoid DILI was not achieved.

In contrast, retigabine (**4**) did not exhibit hepatotoxicity. Instead, bluish discoloration emerged as a unique adverse effects (AEs) in patients treated for at least 2 years [3]. A mechanistic study suggests that the interaction of retigabine with melanin plays a central role in this phenomenon. Melanin binding effectively concentrates retigabine molecules and brings them into close proximity, enabling condensation reactions under the necessary oxidative conditions. Initial oxidation of retigabine yields quinone diimine intermediates **6** (X═CH). These intermediates undergo dimerization and subsequent aromatization, yielding phenazinium cationic species **8,** responsible for the observed bluish discoloration [41]. A similar formation of flupirtine dimers is highly unlikely as the N‐atom of the pyridine ring would have to form a second positively charged atom directly adjacent to the cationic center of the pyrazinium structure.

Given the structural similarity, azetukalner, which contains an aminoanilide core, raises a plausible concern for analogous metabolic activation and quinone diimine formation. It was designed to bypass the pigment‐forming pathway associated with retigabine [42]. Unlike retigabine, which bears a secondary aromatic amino group, the amino functionality of azetukalner is incorporated within a tetrahydroisoquinoline ring system, which sterically and electronically constrains the nitrogen atom and prevents **1** from participating in oxidative dimerization.

**Scheme 3:**
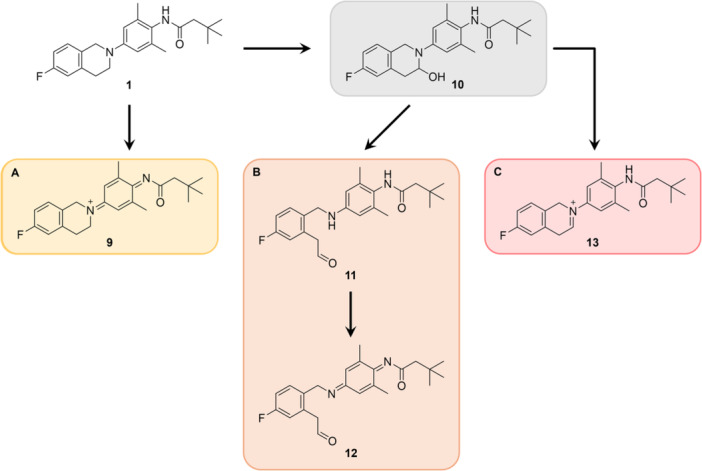
Three hypothetical metabolic pathways of azetukalner to form reactive metabolites. (A) Direct oxidation of the diaminobenzene core to quinone diimines **9**. (B) CYP‐mediated α‐hydroxylation of the tetrahydroisoquinoline ring forming a hemiaminal **10** and subsequent hydrolytic ring opening to yield a secondary aminoanilide **11** prone to oxidation to quinone diimines **12**. (C) Dehydration of the same hemiaminal **10** to form an electrophilic dihydroisoquinolinium ion **13**.

However, the inability of azetukalner to form retigabine‐like dimers does not necessarily preclude the risk of forming reactive metabolites. Thus, three potential bioactivation pathways merit consideration (Scheme 3).

First, the central aromatic ring of azetukalner is still a 1,4‐disubstituted diaminobenzene. One of the two remaining N‐atoms adjacent to the central core bears the pivaloyl amide, and the other is embedded in a tetrahydroisoquinoline ring. As with paracetamol (USAN acetaminophen), such anilide structures can undergo direct ring oxidation without prior *N*‐deacylation. Similarly, it is possible that the tertiary N‐arylamines could be oxidized to form quinone imines (Scheme 3A), a mechanism observed in compounds such as nefazodone (not shown) [43].

Second, CYP‐mediated hydroxylation at the α‐carbon atom of the tetrahydroisoquinoline ring could theoretically produce an unstable hemiaminal **10**. In theory, this intermediate might undergo hydrolytic ring opening to form a secondary aminoanilide **11**. This intermediate, bearing an abstractable proton, would be susceptible to oxidation to form quinone diimines **12** (Scheme 3B). It should be noted, however, that the existing literature does not currently provide examples of this full metabolic sequence for any N‐containing alicyclic substrates.

Third, and perhaps most likely, is the pathway involving the dehydration of hemiaminal **10** to yield an endocyclic iminium ion **13**. This two‐electron oxidation generates an electrophilic dihydroisoquinolinium species that retains the integrity of the ring system (Scheme 3C). This reactive intermediate is capable of alkylating nucleophilic residues on proteins and can be trapped in vitro using cyanide. This pathway has been experimentally validated for *N*‐methyltetrahydroisoquinoline derivatives, such as nomifensine (not shown), where the corresponding dihydroisoquinolinium metabolite is formed via enzymatic oxidation by cytochrome P450s, monoamine oxidase A, and myeloperoxidase [44, 45]. However, these are just intellectual concerns, and there is no published data available on hypothetical species **13**.

**Figure 2:**
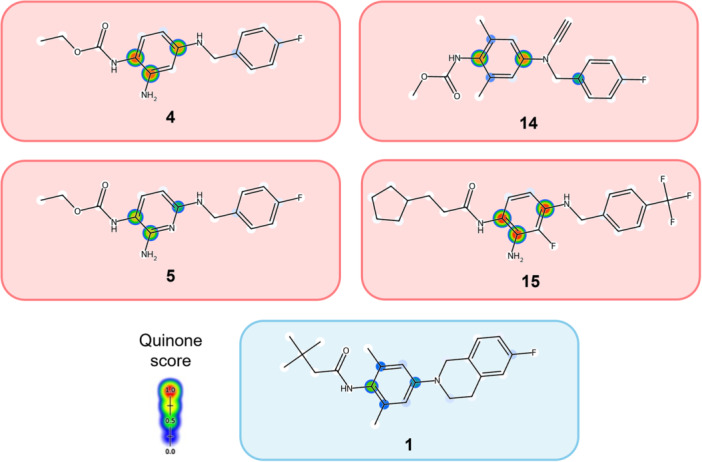
Prediction of quinonation risk using XenoSite [40] reveals high susceptibility for retigabine (**4**), flupirtine (**5**), pynegabine (**14**), and QO‐83 (**15**). In contrast, azetukalner (**1**), exhibits a reduced quinonation risk, approaching the 0.5 threshold, indicating a safer metabolic profile.

To further evaluate the risk of forming reactive quinon metabolites, the quinonation potential of various K_V_7 channel openers was assessed using Xenosite's predictive modeling platform (Figure 2) [46]. This computational analysis revealed that the two formerly marketed K_V_7 activators, **4** and **5**, exhibit significantly elevated quinone formation scores, consistent with their known metabolic pathways involving oxidation and their association with discoloration and liver toxicity, respectively. Other promising candidates, such as pynegabine **14** and QO‐83 **15**, also fail to mitigate the issue of reactive metabolite generation, as evidenced by high predicted quinone formation scores. In contrast, azetukalner, specifically designed to avoid dimerization through structural modification, showed a markedly reduced quinonation score approaching 0.5, the established threshold for significant quinone formation. While these results are based on a predictive model rather than experimental metabolism studies, the low score suggests a potentially lower likelihood of generating toxic quinoid species compared to earlier agents in this class.

Many drugs contain toxifiable metabophores, yet their clinical relevance is often prevented by the liver's robust detoxification capacity, embodying the well‐known principle that “the dose makes the poison”. This is evident in the contrasting profiles of retigabine (600–1200 mg/day) and azetukalner (25 mg/day), where the significantly lower dose of azetukalner may reduce the burden on detoxification pathways. Besides, the notoriously problematic primary amino group of both flupirtine and retigabine was not associated with toxic effects in the clinic. However, another advantage of azetukalner is that this potential metabolic vulnerability could be replaced by a methyl group.

## 5 Structure–Activity Relationships

**Figure 3:**
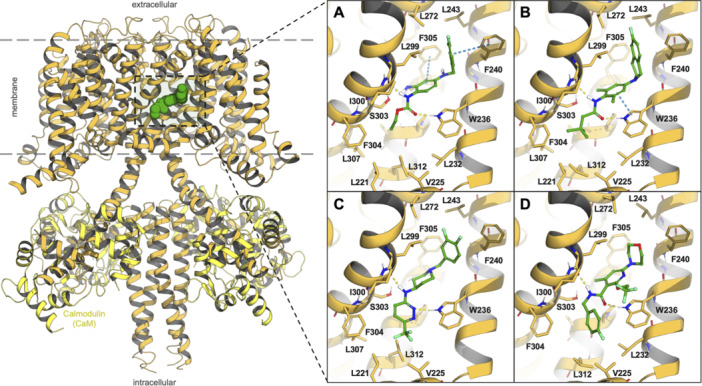
Overview of the K_V_7.2 structure in complex with calmodulin (left), including the binding mode of retigabine (A) as determined by cryo‐EM (PDB: 7CR7) [47]. Predicted binding modes, obtained via induced fit docking (Glide) [48, 49, 50], are shown for azetukalner (B), JNJ‐37822681 (C), and compound 18c reported by Wurm et al. (D) [51]. Hydrogen bonds and π–π interactions are indicated by yellow and cyan dashed lines, respectively.

The membrane‐facing modulator‐binding site within the pore domain of K_V_7 potassium channels is a well‐established pharmacological hotspot (Figure 3). This site is highly conserved across the K_V_7.2—K_V_7.5 subtypes and is characterized by a critical tryptophan residue that serves as a key interaction partner for most known modulators. Structural insights into this region have primarily been derived from cryo‐EM studies. However, to date, only homotetrameric structures of K_V_7.2, K_V_7.4, and K_V_7.5 in complex with various modulators have been resolved, and no experimental structure for azetukalner is publicly available. To address this limitation, we used the K_V_7.2 structure in complex with retigabine as a template to predict the binding mode of azetukalner, along with two additional K_V_7 modulators for comparison. Induced fit docking was performed using Glide to account for receptor flexibility and ligand‐induced conformational adjustments. For clarity and consistency, residue numbering throughout this section refers to K_V_7.2.

Notably, the residues directly involved in ligand binding are highly conserved. In K_V_7.2/7.3 heterotetramers, the interacting residues are identical to those in K_V_7.2 homotetramers. Even in K_V_7.4 and K_V_7.5, only a single residue differs within the binding site. This high degree of conservation complicates the achievement of subtype selectivity through direct binding interactions alone and provides a structural rationale for the modest selectivity observed for azetukalner, which is less than one order of magnitude.

The predicted binding mode of azetukalner closely resembles that of retigabine (Figure 3A). The amide group forms two key hydrogen bonds (Figure 3B): one with W236 (a hydrogen‐bond acceptor) and another with L299 (a hydrogen‐bond donor). These interactions are considered central to ligand anchoring. In contrast, hydrogen bonds formed by the primary aromatic amine of retigabine with S303 and F305 are not essential and are frequently absent or substituted in other modulators. Consistently, in azetukalner, this functional group is replaced by a methyl substituent [52].

An additional methyl group in the ortho position relative to the amide further restricts rotation around the amide bond. This conformational constraint leads to entropically favorable preorganization of the ligand, a strategy previously described for related compounds. A second entropy‐driven optimization is achieved by reducing the number of freely rotatable bonds in the 4‐fluorobenzylamine moiety. In azetukalner, this is accomplished by cyclization into a tetrahydroisoquinoline substructure, effectively bridging the aromatic ring to the secondary nitrogen atom. Due to the sp^3^ hybridization of the nitrogen atom, this partially saturated ring system can adopt a half‐chair conformation. This conformation positions the substituent nearly orthogonal to the central aromatic ring, similar to morpholine‐containing analogs (Figure 3D), thereby enabling efficient occupation of the upper hydrophobic pocket. On the opposite side of the molecule, the ethyl group present in retigabine is replaced by a bulkier aliphatic substituent in azetukalner, allowing improved filling of the lower hydrophobic pocket.

The central aromatic ring of azetukalner can also engage in π–π interactions with W236 and F305. Regardless, these interactions appear to contribute only marginally to overall binding affinity. Supporting this observation, compounds such as JNJ‐37822681 lack a central aromatic ring altogether (Figure 3C). However, such modifications tend to increase the basicity of nitrogen atoms, leading to protonation under physiological conditions and, consequently, reduced membrane permeability. In contrast, azetukalner exhibits a calculated pKa (Epik [53]) of approximately 3.7 for its conjugated acid, indicating that it remains largely unprotonated at physiological pH. This physicochemical property likely contributes to improved membrane penetration and supports its pharmacological profile.

Retigabine and related K_V_7 modulators have been shown to stabilize the open state of K_V_7 channels through mechanisms functionally linked to Phosphatidylinositol‐4,5‐bisphosphate (PIP_2_) dependence. Although such a mechanism has not yet been comprehensively demonstrated for Azetukalner, its structural and pharmacological similarities to other K_V_7 modulators may indicate the possibility of a comparable PIP_2_‐dependent mode of action [54, 55].

## 6 Clinical Data

### 6.1 Phase I

The safety, tolerability, and pharmacokinetics (PK) of XEN1101 were evaluated in Phase I clinical trials. To provide early readouts of pharmacodynamic effects and indirect evidence of CNS delivery, transcranial magnetic stimulation (TMS) measurements were incorporated into a study of healthy volunteers. By utilizing the resting motor threshold (RMT) as a biomarker for corticospinal excitability and TMS‐evoked potentials for cortical excitability, researchers demonstrated that XEN1101 significantly reduces both corticospinal and cortical excitability. Following administration, the RMT increased in a time‐dependent manner, which correlated with plasma concentrations of XEN1101.

Single‐ and multiple‐ascending‐dose studies were conducted in healthy volunteers to evaluate PK and tolerability, including an evaluation of food effects. Testing included single doses of 5 mg, 15 mg, 20 mg, and 30 mg, as well as multiple doses of 15 mg once daily for 7 days. Key PK findings revealed peak plasma concentrations (C_max_) of 31.5 ng/mL (20 mg) and 35.5 ng/mL (30 mg), with corresponding half‐lives of 41 h and 63.4 h. Elimination followed a biphasic pattern, characterized by a functional half‐life of 1–2 days and a terminal half‐life exceeding 5 days. XEN1101 was generally well tolerated. Reported adverse events were mild‐to‐moderate and transient, primarily consisting of CNS‐related effects such as drowsiness and dizziness. No serious adverse events were reported [34, 36, 56, 57, 58].

Further clinical evaluation included a trial focused on the mass balance, recovery, and metabolite profiling of radiolabeled ^14^C‐XEN1101 [35].

### 6.2 Phase II

Azetukalner was evaluated in two Phase II trials, namely X‐TOLE and X‐NOVA.

The X‐TOLE Phase II clinical trial was designed as a randomized, double‐blind, placebo‐controlled, multicenter study with an optional OLE to evaluate the clinical efficacy, safety and tolerability of azetukalner administered as adjunctive treatment in adult patients aged 18 to 75 years diagnosed with focal epilepsy. 325 adults with focal‐onset seizures were enrolled. During the double‐blind period (DBP) of 8 weeks, patients either received once daily azetukalner (10 mg, 20 mg, or 25 mg) or placebo according to randomization. The main objective was the median percent change (MPC) in monthly focal seizure frequency versus placebo. All active doses demonstrated significant, dose‐dependent reductions in seizure frequency compared to placebo 52.8%, 46.4%, and 33.2% reductions at 25 mg, 20 mg, and 10 mg, respectively. The drug was well tolerated, and 88% completed the trial [32, 59, 60].

The X‐TOLE participants who completed the DBP with a minimum of 80% study medication compliance and no important protocol deviations were eligible to enter the OLE phase, during which participants from all DBP arms began treatment with azetukalner 20 mg taken once daily with food. Interim analysis of the ongoing OLE study following X‐TOLE was published in February 2025. The data cut‐off was 2 years after the completion of the DBP of X‐TOLE. 165 participants, 60.0% of the original 275 OLE enrollees, were treated for 24 months. Response rates for all OLE Participants (n = 275) were 50% seizure reduction 44.4%, 90% seizure reduction 19.6%, and seizure freedom achieved 14.9% for 12 consecutive months. Long‐term treated patients (24 months, n = 165) showed enhanced results, including 50% seizure reduction 69.7%, 90% seizure reduction 31.5%, and seizure freedom in 23.6% of long‐term patients for 12 consecutive months. Adverse events occurred in 87.3% of participants, with dizziness, headache, and somnolence being the most frequent. Serious adverse events (SAEs) occurred in 12.7% of participants. The most frequent SAEs were changes in seizure presentation (2.2%), followed by paresthesia, seizure, pneumonia aspiration, deep vein thrombosis, and falls (each 0.7%). One death occurred but was determined to be unrelated to azetukalner. Treatment discontinuation due to AEs occurred in 10.9% of patients, driven primarily by dizziness and somnolence, along with amnesia. Other neurological events of interest included gait disturbance, aphasia, and confusional state. Compared to retigabine, the SAE rate for azetukalner falls within a similar range of 4%–27%. Both agents share a similar spectrum of neurological events, such as dizziness, somnolence, headache, gait disturbances, confusional state, and fatigue, which may contribute to balance disorders or falls. However, azetukalner lacks the recognizable pattern of drug‐induced organ toxicity, specifically the urinary retention and ophthalmological abnormalities (e.g., retinal pigmentation and visual acuity changes), that limited the clinical use of retigabine [9, 61].

The X‐NOVA phase II clinical trial was a randomized, double‐blind, parallel‐group multicenter, proof‐of‐concept study. In total, 168 adults with moderate to severe MDD were enrolled. Patients were equally randomized and received 10 mg, 20 mg, or a placebo once daily for the DBP of 6 weeks. Concomitant antidepressant medications were not permitted. The study did not meet its primary endpoints as the reduction in depression score (MADRS) at week 6 for the 20 mg dose was not statistically significant versus placebo (3.04 points, *p* = 0.14). Despite this, some exploratory signals were observed, including early symptom improvement at week one and reductions in Hamilton depression scale and anhedonia scores (SHAPS). AEs were generally mild and well‐tolerated across treatment groups [33, 59, 62].

Another randomized, double‐blind, placebo‐controlled phase II trial evaluated azetukalner in individuals with MDD and anhedonia. The study population included 60 participants aged 18 to 65 with MDD and clinically significant anhedonia (SHAPS 20). During DBP, patients received either 20 mg azetukalner daily or a placebo for 8 weeks. As the primary endpoint, they wanted to observe changes in bilateral ventral striatum activity during reward anticipation via functional magnetic resonance imaging. This study also failed to meet its primary endpoint, showing no significant difference in ventral striatum response between groups. Improvements in MADRS and SHAPS scores were noted but did not reach statistical significance. AEs are consistent with prior observations [31, 63].

While these data suggest a potential role for azetukalner in treating MDD, the lack of consistency in meeting primary endpoints indicates that robust evidence of efficacy has not yet been established.

### 6.3 Phase III

The X‐TOLE2 Phase III clinical trial was a multicenter, randomized, double‐blind, placebo‐controlled study evaluating azetukalner as adjunctive therapy in adult patients with treatment‐resistant focal onset seizures. A total of 374 patients with highly refractory epilepsy were enrolled and randomly assigned to receive either 25 mg azetukalner, 15 mg azetukalner, or placebo once daily, with no titration required. Most patients had a history of polypharmacy, with multiple concomitant antiseizure medications. Following a baseline period, a 12‐week double‐blind treatment phase commenced, followed by an optional OLE.

The primary objective was to assess the effect of azetukalner versus placebo on MPC in monthly focal onset seizure frequency. Secondary objectives included evaluation of responder rates at least 50% seizure reduction, early treatment effects at week one, and improvements in patient and clinical global impression of change (PGI‐C and CGI‐C) scores, reflecting subjective and clinician‐rated health improvements.

**Figure 4:**
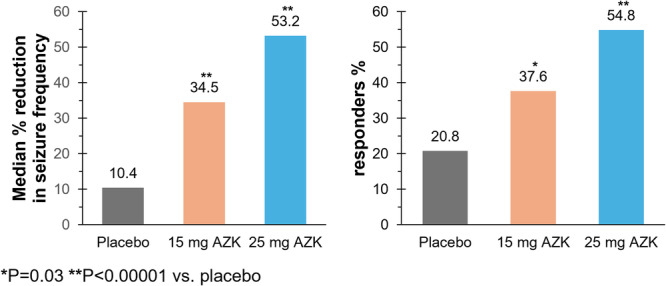
Median reduction in focal seizure frequency (%) and responder rates (50% seizure reduction) in patients from the X‐TOLE2 clinical trial of azetukalner (AZK) in focal‐onset seizures. Data sourced from Xenon [64].

According to topline results reported by Xenon Pharmaceuticals, which have not yet been published in a peer‐reviewed journal, the 25 mg dose was associated with a reduction of 53.2% in monthly seizure frequency, compared with a 10.4% decrease in the placebo group (*p* < 0.000000000006), yielding in a placebo‐adjusted reduction of 42.7%, exceeding the results of the X‐TOLE phase 2 trial. The 15 mg dose also demonstrated a statistically significant reduction in seizure frequency of 34.5%, suggesting a dose‐dependent response. Sponsor‐reported data further indicate that both doses improved responder rates compared with placebo (Figure 4). The 25 mg dose shows a statistically significant reduction as early as week 1. Additionally, improvements were noted in PGI‐C and CGI‐C scores.

Azetukalner was generally well‐tolerated, particularly at the 15 mg dose. However, the 25 mg dose was associated with a higher rate of treatment‐emergent AEs, resulting in a higher discontinuation rate of 14.5% compared with 3.2% for placebo. The most common AEs in the 25 mg group included dizziness (20.5%), somnolence (8.8%), headache (8.8%), and fatigue (7.6%). Serious adverse events were rare and evenly distributed across groups, with no reported instances of severe cutaneous reactions such as Stevens–Johnson syndrome or drug reaction with eosinophilia and systemic symptoms (DRESS).

Based on the preliminary results, the 25 mg dose appears to provide significant seizure suppression and rapid onset of action in refractory populations. However, the higher discontinuation rate associated with CNS‐related side effects suggests that the 15 mg dose may offer a more favorable balance of efficacy and tolerability [23, 64].

## 7 Conclusion

The development of azetukalner demonstrates that the problematic safety profiles of flupirtine and retigabine can be successfully decoupled from their therapeutic efficacy. Its progression toward a New Drug Application indicates that a clinically viable, safer follow‐up for the K_V_7 class is achievable, thereby reopening therapeutic avenues for focal epilepsy.

Moreover, a safer, registered pharmaceutical ingredient may revitalize ongoing research in areas where retigabine was limited to use as a pharmacological tool, including major depression [65], multiple sclerosis [66, 67], and dementia following repetitive mild brain trauma [68]. By overcoming previous safety barriers, azetukalner enables a comprehensive exploration of indications linked to the normalization of ion channel function and the restoration of blood‐brain barrier integrity.

However, while azetukalner represents a significant milestone in pharmacological redesign, it is more likely a stepping stone than the final destination in K_V_7 drug development. While it successfully mitigates the metabolic liabilities of its predecessors and widens the therapeutic window, further optimization is required to achieve a more refined subtype‐selectivity profile. Specifically, the ability to distinguish between CNS‐predominant (K_V_7.2/3) and periphery‐predominant (K_V_7.4/5) subtypes is essential to minimize systemic adverse effects.

Given the minimal residue variation at the binding site utilized by retigabine and azetukalner, achieving significant subtype selectivity via traditional iterative optimization is likely precluded by the inherent conservation of the binding pocket. Consequently, the most viable strategy lies in the discovery of novel chemotypes that target alternative binding pockets. Compounds such as E0714 represent a promising shift, as targeting distinct sites may bypass the limitations of the conserved retigabine‐binding pocket and provide a more refined selectivity profile [69].

Finally, the transition toward precision pharmacology remains a priority. For pediatric patients with KCNQ‐associated developmental and epileptic encephalopathies, the development of compounds targeting specific gain‐of‐function or loss‐of‐function mutations would be transformative. Whether through the repurposing of existing agents or the design of novel subtype‐selective modulators, the future of this class depends on evolving from broad activation to selective or mutation‐specific precision.

## Conflicts of Interest

The authors declare no conflicts of interest.

## Data Availability

Data sharing is not applicable to this article as no datasets were generated or analyzed during the current study.
